#  Synthesis and Antibacterial Activity of Novel Curcumin Derivatives Containing Heterocyclic Moiety 

**Published:** 2013

**Authors:** Othman A. Hamed, Noha Mehdawi, Adham Abu Taha, Emad M. Hamed, Mohammed A. Al-Nuri, Ayman S. Hussein

**Affiliations:** a *Department of Chemistry, An-Najah National University, Nablus, West Bank, Palestine.*; b *Department of Chemistry, Hashemite University, Zarqa, Jordan.*; c *College of Pharmacy, Genetics Laboratory, An-Najah National University, Nablus, West bank, Palestine. *

**Keywords:** Antimicrobial activity, pyrazoles, isoxazoles, diazepine, disc diffusion

## Abstract

A series of curcumin derivatives containing heterocyclic moiety have been synthesized and evaluated for their antibacterial activities. The chemical structures of the synthesized compounds were verified on the basis of spectral data and elemental analyses. Investigation of antimicrobial activity of the derivatives demonstrated the ability to inhibit Gram-positive microorganisms with zone of inhibition ranging from 14-18 mm, MIC ranging between 0.0625 and 0.25 mg/mL. Among all tested derivatives, diazepine 4 exhibited remarkable potency against Gram-positive bacteria *S. aureus. *An extensive study is underway to optimize the effectiveness of diazepine type of compounds and to determine their mode of action.

## Introduction

Bacterial resistance to many available antibacterial agents is a growing problem. Accordingly, the development of new antibacterial agents that could overcome the resistance problem has become the subject of an ongoing research (1-6). In the present work, we have employed curcumin as a naturally occurring skeleton for the construction of heterocyclic systems such as pyrazole, isoxazole, and diazepine which might exhibit promising antibacterial activity. 

Curcumin, (E,E)-l,7-bis(4-hydroxy-3- methoxyphenyl)-l,6-heptadiene-3,5- dione, also known as turmeric yellow, is a natural yellow pigment derived from the roots of curcuma plants*, e.g. *C. tinctoria, C. xanthorrhiza and C. domestica, and is known since several hundred years. It is used as a food coloring agent and in traditional Indian medicine for treatment of various diseases that include biliary disorders, anorexia, cough, diabetic wounds, hepatic disorder, rheumatism, blood purification and rheumatoid arthritis (7-10). 

Several studies have shown that curcumin has various pharmacological activities including potent antioxidant, anti-inflammatory and antiviral activities (11-16), as well as anticancer activities against different forms of cancer, *e.g*., cervical cancer caused by HPV (17-19). In addition, other studies have shown that curcumin represents a hopeful approach for delaying or preventing the progression of Alzheimer›s disease (20-24), and has been identified as an inhibitor of HIV-1 LTR directed gene expression and viral replication, besides its ability to block HIV replication by inhibiting HIV-integrase and protease (25-30). Heterocyclic compounds, in general, are very important class of organic compounds with various bioactivities ranging from antibacterial to anticancer (31-47). For example, diazepines and benzodiazepines have various therapeutic applications. Many members of the diazepine family are widely used as anticonvulsants, anti-anxiolytics, analgesics, sedatives, antidepressants and hypnotic agents (48-51). These literature findings have led us to synthesize the proposed group of curcumin-based heterocycles and screen them against representative panel of Gram-positive and Gram-negative bacteria. 

## Results and Discussion

Compounds used in this study were prepared following a literature procedure with minor modification (52-54). In this procedure, curcumin (compound 1) was refluxed with various hydrazines in acidic media, which performs dual function as catalyst and solvent. Acid used for this purpose was either glacial acetic acid or polyphosphoric acid. The progress of the reaction was monitored by TLC. Some reactions required more reflux time than the others. Followed procedure produced only the expected product; some starting materials were also observed in some reactions, as in the case of synthesis of 1-(2,4-dinitrophenyl)-3,5-bis-2-(4-hydroxy-3- methoxystyryl)-1H-pyrazole (2b). Similar yields were obtained using either acetic acid or PPA as a solvent. Purification of the products was performed using flash chromatography. Purified products were analyzed by various analytical and spectroscopic techniques, such as: melting point, LC/MS, ^1^H and ^13^C NMR, and elemental analysis. In all cases, results are consistent with the expected structures. All compounds were obtained in acceptable yield (65 % to 90%). 

**Figure 1:**
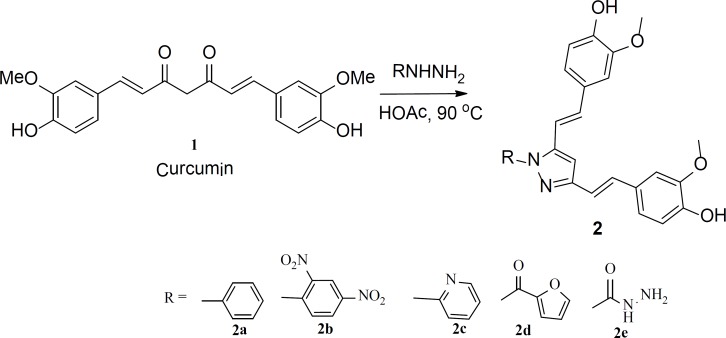
Curcumin based pyrazoles prepared form reacting curcumin with various hydrazines

The structures and the characterization data for the prepared compounds are summarized in Figure 1 and the experimental part, respectively. Another set of curcumin-based heterocycles was prepared by reacting curcumin with various amines such as ethylenediamine and hydroxylamine. Compound 5 was prepared from reacting curcumin with *n*-butylamine; it was added to the set of evaluated derivatives for a comparison purpose. The structures and the characterization data for these compounds are also summarized in Figure 1 and the experimental part, respectively. 

*Antibacterial activity*

The *in-vitro *antimicrobial activity was performed on four types of bacteria strains: *S. aureus*, *E. coli*, *Proteus mirabilis *and *Pseudomonas aeruginosa *using a disk diffusion assay reported in the literature by Perez *et al. *(55). All strains were isolated from patients suffering from bacterial infections with the relevant bacteria. Ampicillin, Methicillin, and Carbenicillin were used as references of antibacterial compounds in antimicrobial assay.

*Screening results*

The results of the prepared compounds (2a-e, 3, 4, 5) and the references for preliminary antibacterial testing are shown in Table 1. Results revealed that the majority of the synthesized compounds showed varying degree of inhibition against the tested microorganisms. In general, the potency against Gram-positive organisms is greater than against Gram-negative ones. As shown in Table 1, curcumin and all other derivatives were inactive (zones of inhibition were zero) against *Proteus mirabilis *and *Pseudomonas aeruginosa*. However, some compounds showed potency against *E. coli*, and some others like curcumin, compounds 4, and compound 5 were totally inactive against *E. coli*. Compound 2b showed the highest activity against *E. coli *(zone of inhibition is about 13 mm)**.**

**Table 1:** Zone of Inhibition of Curcumin and Synthesized Curcumin-Based Heterocycles

**Compound**	**Gram-positive **Bacteria ***S. aureus***	**Gram-negative **Bacteria		
		***P. mirabilis***	***E. coli***	***P*** **. ** ***aeruginosa***	
**Curcumin (1)**	11	0	0	0
**Ampicillin**	25	21	18	NT^a^
**Methicillin**	16	NT^a^	NT^a^	NT^a^
**Carbenicillin**	15	22	23	20
**2a**	11	0	11	0
**2b**	14	0w	13	0
**2c**	16	0	10	0
**2d**	15	0	12	0
**2e**	18	0	12	0
**3**	16	0	12	0
**4**	27	0	0	0
**5**	11	0	0	0

All prepared curcumin-based heterocycles have shown to be susceptible to excellent potency against Gram-positive bacteria S*. aureus *with zone of inhibition ranges from 11 mm to 27 mm. The results were comparable to those of the reference antibiotics Methicillin, and Carbenicillin. Compound 4, showed remarkable activity against S*. aureus *with zone of inhibition of about 27 mm and its effect is about two-fold more than that of some other derivatives. Compound 4 showed even higher potency against S*. aureus *than Ampicillin (Table 1). Curcumin-based heterocycles then subjected to minimum inhibition concentration (MIC) testing. Results on *S. aureus *bacteria are summarized in Tables 2 and 3. As shown in Table 2, MIC of curcumin was 1 mg/mL, which is at least four-fold higher than the MIC of the other curcumin derivatives. Compounds 2a, 2b and 3 showed MIC of about 0.25 mg\mL. Derivatives 2c, 2d, 2e, and 5 showed lower MIC (about 0.0625 mg/mL). These results indicate that compounds 2c, 2d, 2e, and 5 have higher activities than compounds 1, 3, and 5. However, the highest activity was shown by derivative 4 as it showed a MIC of 1.9 μg mL^-1^**.**

**Figure 2:**
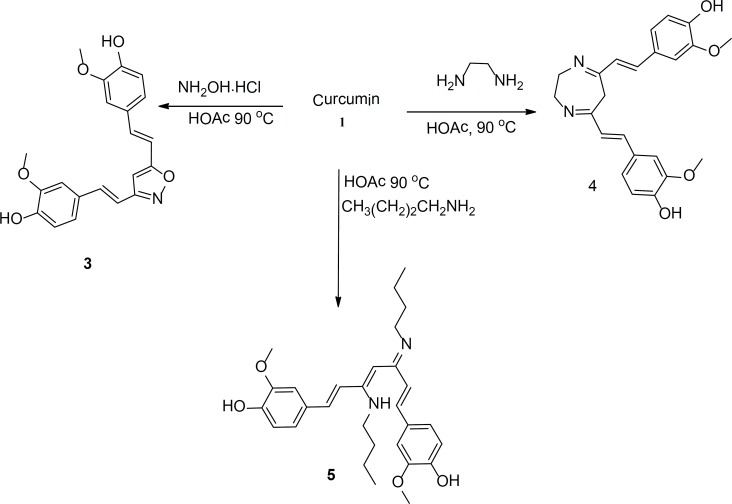
Curcumin based isoxazoles, diazepine, and amine prepared form reacting curcumin with various amines.

**Table 2:** MIC of curcumin and synthesized Curcumin-Based Heterocycles on *S. aureus *Bacteria

**Conc.(μg/mL)**	**Curcumin**	**2a**	**2b**	**2c**	**2d**	**2e**	**3**	**4**	**5**
**4 x 10** ^3^	-	-	-	-	-	-	-	-	-
**2 x 10** ^3^	-	-	-	-	-	-	-	-	-
**1 x 10** ^3^	-	-	-	-	-	-	-	-	-
**500**	+	-	-	-	-	-	-	-	-
**250**	+	-	-	-	-	-	-	-	-
**125**	+	+	+	-	-	-	+	-	-
**62.5**	+	+	+	-	-	-	+	-	-
**31.25**	+	+	+	+	+	+	+	-	+
**15.625**	+	+	+	+	+	+	+	-	+
**7.8125**	+	+	+	+	+	+	+	-	+
**3.9062**	+	+	+	+	+	+	+	-	+
**1.953125**	+	+	+	+	+	+	+	-	+
**0.976563**	+	+	+	+	+	+	+	+	+
**Positive control**	+	+	+	+	+	+	+	+	+
**Sterility control**	-	-	-	-	-	-	-	-	-

 The minimum concentration of each curcumin derivatives that results in a total inhibition of bacterial growth (MBC) was also determined. Compound 4 showed the highest activity. MBC for compound 4 was 0.0075 mg\mL. These results indicate that compound 4 has the highest potency against *S. aureus*, which may be attributed to the presence of 1,4-diazepine ring. However, compound 5 that lacks heterocyclic system showed the least potency against *S. aureus *and no activity against *E-Coli*, which is similar to that of curcumin. These results indicate that the improved potency of the prepared compounds could be attributed to the heterocyclic part of the curcumin-based heterocycles. 

## Conclusion

We have synthesized series of curcumin-based heterocycles and evaluated their antibacterial activities against Gram-positive and Gram-negative bacteria. The molecules 2b-e and 3 effectively inhibit *S. aureus*, with zone of inhibition ranging from 14 to 18 mm, MIC ranging between 0.0625 and 0.25 mg/ mL. Among the prepared derivatives, diazepine (4) showed the highest potency against Gram-positive bacteria *S. aureus*. In conclusion, more extensive study is needed to optimize the effectiveness of diazepine type of compounds and to determine their mode of action. This could be accomplished by preparing a variety of curcumin-based diazepine hybrids and screen their antibacterial activities. 

## Experimental

*General experimental*

All chemicals were purchased from Aldrich Chemical Company and used without any further purification unless otherwise specified. All prepared compounds were characterized by ^1^H-NMR, ^13^C-NMR, IR spectroscopy, elemental analysis, and melting point. Nuclear Magnetic Resonance spectra were recorded on Varian Gemini 2000, 300 MHz instrument and on Bruker DPX-300 MHz instruments. Infrared spectra were recorded in KBr on a Shimadzu 820 PC FT-IR spectrometer. All ^1^H-NMR experiments were reported in *δ *units, parts per million (ppm) downfield from tetramethylsilane (TMS). All ^13^C-NMR spectra were reported in ppm relative to deuterochloroform (77.0 ppm). All melting points were determined in an open capillary tube and are uncorrected. At least two measurements were carried out for each compound. Elemental analyses were carried out with Elementar Vario EL III elemental analyzer. TLC analysis was performed on silica gel plates pre-coated with Merck Kieselgel 60 F254 and visualization was done using UV lamp. Sample purifications were performed using flash chromatography with silica gel (100-200) mesh.

*General Procedure*

*Preparation of curcumin-based heterocyclic compounds*

The general experimental procedure for the preparation of compounds shown in Figure 1 was as follows: In a round bottom flask equipped with magnetic stirring bar and a condenser, curcumin (1.5 mmole, 0.5 g) was dissolved in acetic acid (or polyphosphoric acid (PPA)) (8 mL). The desired hydrazine (1.5 mmole) or amine was added to the solution of curcumin in acid. The produced solution was refluxed for 6-12 h. The progress of the reaction was monitored by TLC. After complete conversion, the mixture was cooled to room temperature and concentrated *in-vacuo *and redissolved in ethyl acetate (150 mL). The ethyl acetate layer was washed with saturated solution of NaHCO_3_ and saturated solution of NaCl, dried over Na_2_SO_4_ and concentrated *in-vacuo*. The produced solid was collected by suction filtration and purified by flash chromatography (hexane : EtOAC (6 : 4)). In case where PPA was used, reaction mixture was diluted with water, extracted with ethyl acetate and then the product was purified as shown before.

*1-phenyl-3,5-bis-2-(4-hydroxy-3-methoxystyryl)-1H-pyrazole (2a)*

Curcumin (1.5 mmole, 0.5 g) was dissolved in glacial acetic acid (8.0 mL) and then, phenyl hydrazine hydrochloride (1.5 mmole, 0.25 g) was added. Produced solution was refluxed for about 6 h. Yield 71.6% (0.48 g), mp 127-129°C, IR (KBr ): *v*_max_ cm^-1^ 3600 (-C-OH), 3350 (–C–NH), 3100, 1620 (-C=N), 1600, and 1080 (C-O ether) of (–O-CH_3_). ^1^H-NMR (300 MHz, CDCl_3_) *δ *ppm: 3.90 (s, 6H, OCH_3_), 6.05 (s, 2H, OH), 6.80 (s, 1H, C4-H), 7.02 (d, 2H, J = 14.8 Hz, C2-H and, C6-H), 7.10 (d, 2H, *J *= 14.8 Hz, C1-H and C7-H), 7.15–7.32 (m, 8H, Ar-H), 7.45 (m, 2H, Ar-H), 7.72 (m, 1H, Ar-H). ^13^C NMR (300 MHz, CDCl_3_) *δ *ppm: (300 MHz, CDCl_3_): 56.05, 56.09, 101.22, 110.02, 110.98, 112.57, 115.97, 116.14, 117.64, 120.67, 125.24, 128.17, 128.29, 128.84, 129.02, 129.06, 129.08, 131.22, 133.34, 139.72, 142.82, 147.20, 147.74, 148.2, 148.33, 151.54, 161.00. Anal. Calcd for C_27_H_24_N_2_O_4_: C 73.62, H 5.49, N 6.36. Found: C 73.43, H 5.42, N 6.48.

*1-(2,4-dinitrophenyl)-3,5-bis-2-(4-hydroxy-3-methoxystyryl)-1H-pyrazole (2b)*

Curcumin (1.5 mmole, 0.5 g) was dissolved in glacial acetic acid (8.0 mL), and to it was added 2,4-dinitrophenyl hydrazine hydrochloride (1.5 mmole, 0.355 g). Produced solution was refluxed for about 12 h (Yield 55 (0.43 g), mp 192-195°C).

IR: *v*_max_ cm^-1^ 3610 (-C-OH), 3050, 1635, (-C=N), 1590, 1080 (C-O ether), 1520, 1350.

^1^H-NMR (300 MHz, CDCl_3_) *δ *ppm: 3.78 (s, 3H, OCH_3_), 3.80 (s, 3H, OCH_3_), 6.16 (s, 2H, OH),

6.80 (s, ^1^H, C-4H), 6.83 (m, 4H, C1-H, C2-H, C6-H, and, C7-H), 6.90 (m, ^1^H, Ar-H), 7.10 (d, 2H, Ar-H), 7.20 (d, 2H, Ar-H), 7.40 (d, 1H, Ar-H), 8.00 (d, 1 H, J = 9.43 Hz, Ar-H containing NO2), 8.43 (dd, 1H, J = 2.64 Hz, 9.43 Hz, Ar-H containing NO_2_), 8.90 (d, ^1^H, J = 2.64 Hz, Ar-H containing NO_2_). ^13^C NMR (300 MHz, CDCl_3_) *δ *ppm: 56.15, 56.23, 102.07, 110.00, 110.94, 111.28, 111.72, 116.11, 116.89, 121.59, 121.86, 123.63, 126, 83, 127.91, 128.43, 128.92, 130.23, 133.07, 135.18, 135.69, 147.16, 144.89, 145.48, 146.23, 148.23, 148.42, 154.02. Anal. Calcd for C_27_H_22_N_4_O_8_: C 61.13, H 4.18, N 10.56. Found: C 61.59, H 4.32, N 10.66.

*1-(2-pyridyl)-3,5-bis-2-(4-hydroxy-3-methoxystyryl)-* ^1^ *H-pyrazole (2c)*

Curcumin (1.5 mmole, 0.5 g) was dissolved in glacial acetic acid (8.0 mL), to it was added 2-hydrazinopyridin (1.5 mmole, 0.164 g). Produced solution was refluxed for 12 h. Yield 89.5 (0.6 g), mp 122-125°C IR: *v*_max_ cm^-1^ 3550 (-C-OH), 3050, 1640, (-C=N), 1590, 1080 (C-O ether), 1520, 1350. ^1^H NMR (400 MHz, DMSO-d_6_) *δ*: 3.78 (s, 6H, 2OCH_3_), 6.13 (2H, OH), 6.65 (s, 1H, C_4_-H), 6.78 (m, 4H), 6.95 (m, 4H), 7.13 (m, 2H), 7.35 (d, 1H), 7.63 (d, 1H), 7.72 (t, 1H), 8.21 (d, 1H). ^13^C NMR (400 MHz, DMSO-d_6_) *δ*: 56.17,101.33, 102.45, 110.135, 110.87, 111.79, 115.56, 116.21, 117.50, 120.62, 122.37, 123.68, 126.69, 127.51, 128.85, 132.38, 132.79, 141.17, 144.02,, 147.81, 148.41, 150.08, 152.20, 163.37, 183.64. Anal. Calcd for C_26_H_23_N_3_O_4_: C 70.73, H 5.25, N 9.50, Found: C 70.62, H 5.27, N 9.56.

**Table 3:** A Summary of MIC And MBC Results of Curcumin and Synthesized Curcumin-based Heterocycles on *S. aureus *Bacteria.

**Compound**	**MIC (mg/mL)**	**MBC (mg/mL)**
curcumin	1	1
2a	0.25	1
2b	0.25	1
2c	0.0625	1
2d	0.0625	1
2e	0.0625	1
3	0.25	1
4	0.0019	0.0075
5	0.0625	

*1-(2-furyl)-3,5-bis((E)-4-hydroxy-3-methoxystyryl)-1H-pyrazol (2d)*

Curcumin (1.5 mmole, 0.5 g) was dissolved in glacial acetic acid (8.0 mL) and to it, was added 2-Furoichydrazide (1.5 mmole, 0.2 g). Produced solution was refluxed for 12 h. Yield 81 (0.52 g), mp 138-141°C, IR: *v*_max_ cm^-1^ 3605 (-C-OH), 3030, 1673 (–C=O amido), 1640 ( -C=N), 1590, 1080 (C-O ether), 1520, 1320 (C-O of the five-member ring). ^1^H-NMR (400 MHz, DMSO-d_6_) *δ*: 3.78 (s, 6H, OCH_3_), 6.01 (s, 2H, OH), 6.50-7.32 (m, 13 H), 7.52 (dd, ^1^H, *J*= 16.15Hz). ^13^C-NMR (400 MHz, DMSO-d_6_) *δ*: 56.03, 99.79, 101.4, 111.74, 112.43, 115.18, 116.01, 116.14, 116.37, 120.56, 121.52, 123.67, 126.74, 128.79, 130.06, 141.18, 146.32, 146.67, 148.32, 148.46, 149.89, 157.70, 183.30. Anal. Calcd for C_25_H_22_N_2_O_5_: C 69.76, H 5.15, N 6.51. Found: C 69.66, H 5.18, N 6.62.

*1-carbohydrazidyl-3,5-bis-2-(4-hydroxy-3-methoxystyryl)-1H-pyrazole (2e)*

Curcumin (1.5 mmole, 0.5 g) was dissolved in glacial acetic acid (8.0 mL), to it was added carbohydrazide (1.5 mmole, 0.135 g). Produced solution was refluxed for 12 h. Yield 88.6 (0.0.56 g), mp 151-154°C, IR: *v*_max_ cm^-1^ 3605 (-C-OH), 3320, 3275, 3190 (–C–NH), 3020, 1635 (-C=N), 1600, 1080 (C-O ether).^1^H-NMR (400 MHz, DMSO-d_6_) *δ*: 3.78 (s, 3H, OCH_3_), 4.32 (s, 2H, NH), 6.01 (s, 2H, OH), 6.50-7.40 (m, 11H), 8.80 (s, H, NH). ^13^C-NMR (400 MHz, DMSO-d_6_): 56.07, 56.12, 100.1, 110.6, 116.08, 116.24,120.30, 120.66, 123.16, 123.34, 128.62, 129.81, 130.4, 131.03, 143.78, 144.70, 147.13, 147.51, 147.50, 150.69, 151.13, 161.06. Anal. Calcd for C_22_H_22_N_4_O_5_: C 62.55, H 5.25, N 13.26. Found: C 62.63, H 5.25, N 13.37.

*3,5-bis-2-(4-hydroxy-3-methoxystyryl)-isoxazole (* 3 *)*

Curcumin (1.5 mmole, 0.5 g) was dissolved in glacial acetic acid (8.0 mL) and to it, was added hydroxylamine hydrochloride (1.5 mmole, 0.11g). Produced solution was refluxed for 12 h. Yield 72.0 (0.4 g), mp 116-119°C, IR: 3570 (-C-OH), 3030, 1605, 1586 (-C=N), 1330 (C-O of the five-member ring) cm^-1^. ^1^H-NMR (400 MHz, DMSO-d_6_): ^1^H-NMR: *δ *3.85 (s, 6H, 2OCH3), 6.25 (s, 2H, OH), 6.71 (s, 1H, C4-H), 6.84-7.01 (m, 3H), 7.04-7.15 (m, 4H), 7.26 (m, 3H). ^13^C-NMR (300 MHz, CDCl_3_) *δ *ppm: 56.14, 56.18, 98.33, 110.56, 110.82, 113.12, 113.43, 115.98, 116.11, 116.24, 121.8, 122.16, 127.49, 127.83, 129.22, 135.27, 135.98, 148.22, 148.42, 162.70, 168.84. Anal. Calcd for C_21_H_19_NO_5_: C 69.03, H 5.24, N 3.38. Found: C 68.89, H 5.21, N 3.41.

*3,5-bis-2-(4-hydroxy-3-methoxystyryl)-3,6-dihydro-2H-1,4-diazepine (* 4 *)*

Curcumin (1.5 mmole, 0.5 g) was dissolved in glacial acetic acid (8.0 mL) and then, ethylenediamine (1.5 mmole, 0.1 mL) was added to the solution. Produced solution was refluxed for 4 h. Yield 76.3 (0.45), mp 65-68°C (hydrated) IR: *v*_max_ cm^-1^ 3605 (-C-OH), 3020, 1640 (-C=N), 1600, 1080 (C-O ether). ^1^H-NMR (400 MHz, DMSO-d_6_) *δ*: 3.16 (s, 2H, CH_2_), 3.764 (s, 4H, CH_2_CH_2_); 3.78 (s, 6H, OCH_3_), 5.72 (s, 2H, OH), 6.85 (m, 2H), 6.91 (d, 2H, J = 15.2 Hz), 7.05 (d, 2H, J = 12.1 Hz), 7.20 (s, 2H), 7.40- 7.60 (m, 2H). ^13^C-NMR (400 MHz, DMSO-d_6_) *δ*: 24.50, 48.30, 56.40, 112.90, 116.30, 120.60, 122.40, 127.80, 129.30, 148.20, 149.30, 165.80. LC/MS [M + 1] for C_23_H_24_N_2_O_4 _Calcd 393.0, found: 394.0. Anal. Calcd for C23H24N2O4: C 70.39, H 6.16, N 7.14. Found: C 70.33, H 6.21, N 7.19. The differences in the [M+1] values could be due to the protonation of compound 4 during LC/MS analysis, since mobile phase used in the analysis was an acidic solution of aqueous methanol.

*4,4›-((1E,3Z,5Z,6E)-3-(butylamino)-5-(butylimino)hepta-1,3,6,triene-1,7-diyl) bis(2-methoxyphenol) (* 5 *)*

Curcumin (1.5 mmole, 0.5 g) was dissolved in glacial acetic acid (8.0 mL) and to it was added *n*-butyl amine (3 mmole, 0.25 mL). Produced solution was refluxed for 10 h. Yield 78% (0.56 g), mp 106-109°C IR: *v*_max_ cm^-1^ 3605 (-C-OH), 3335 (–C–NH), 3040, 1640 (-C=N), 1600, 1080 (C- O ether). ^1^H-NMR (400 MHz, DMSO-d_6_) *δ*: 0.79 (m, 6H), 1.23 (m, 4H), 1.32-1.41 (m, 4H),1.82 (s, 1H), 2.52 (t, 2H), 2.72 (t, 2H), 3.78 (s, 6H, OCH_3_), 6.10 (s, 2H, OH), 6.62 (s, 1H, C_4_-H), 6.84 (m, 4H, C_2_-H, C_6_-H), 7.10 (m, 2H), 7.20 (m, 2H), 7.40 (d, 2H). ^13^C-NMR (400 MHz, DMSO-d_6_) *δ*: 13.60, 13.60, 19.20, 19.20, 35.30, 36.10, 44.50, 46.80, 56.20, 56.43, 97.31, 109.74, 111.95, 115.83, 116.12, 116.11, 121.50, 123.80, 125.31, 129.3, 129.31, 135.55,136.26, 148.23, 148.27, 149.92, 149.93, 152.81, 174.65. Anal Calcd for C_29_H_38_N_2_O_4_: C 72.77, H 8.00, N 5.85. Found: C 72.54, H 8.12, and N 5.76.

*Antibacterial activity of materials*

Culture media: Mueller-Hinton**, **Tryptic Soy Broth was obtained from Hylabs, Israel**.**

*Microorganisms used*

Bacterial strains used in the study were clinical isolates of *Staphylococcus aureus*, *Escherichia coli*, *Proteus mirabilis*, and *Pseudomonas aeroginosa*, were all isolated from patients suffering from bacterial infections with the relevant bacteria.

*Procedure*

These isolates were tested for their susceptibility to the prepared curcumin-based heterocyclics as follows:

Solutions of these derivatives were prepared at concentration of 4 mg per 1 mL of dimethyl sulfoxide (DMSO) solvent, and then incubated for 24 h at 37°C.

*Screening the antimicrobial activity*

The antibacterial bioactivities of the prepared heterocyclics were screened using the well diffusion method reported in the literature by Perez *et al *(55)*.*

Three colonies of bacteria were transferred to sterile tubes each containing 5 mL of Tryptic Soy Broth. Turbidity of the bacterial suspensions was then adjusted to reach an optical density equivalent to a 0.5 McFarland standard to give a bacterial suspension of 10^8^ cfu/mL (cfu: colony forming unit). Mueller-Hinton agar plates were inoculated through streaking bacterial swabs over the entire surface of the plates. Produced Plates were allowed to dry at room temperature and 6 mm wells were punched in each plate. In each plate, 50 μL of 4 mg/mL solutions of each curcumin derivatives were added into duplicate wells. Plates were allowed to stand at room temperature to let the tested derivative be diffused into the agar, and afterwards, they were incubated at 37°C for 18 to 24 h. Then, plates were examined for bacterial growth inhibition and zones of inhibition were measured in millimeters.

*Determination of minimum inhibitory concentration (MIC)*

Two-fold serial dilutions were prepared from the derivative solution in Tryptic Soy Broth. Duplicate tubes of each dilution were inoculated with 5 × 10^5^ of the bacterial strains. All tubes were incubated at 37°C for 18 to 24 h. The highest dilution of the drug that resulted in inhibition of bacterial growth was considered as the MIC.

*Determination of minimal bactericidal concentration (MBC)*

Subcultures from the above dilutions were done on Muller-Hinton plates and incubated at 37°C for 18 to 24 h. The highest dilution that resulted in total inhibition of bacterial growth was then determined and considered as MBC.
